# Review of Bleeding and Thrombotic Risks Associated With Antithrombotic Therapy After Transcatheter Structural Heart Interventions

**DOI:** 10.1016/j.jacasi.2023.08.004

**Published:** 2023-10-24

**Authors:** Hideyuki Kawashima, Yohei Numasawa, Naoki Hayakawa, Taku Asano, Shigemitsu Tanaka, Sho Torii, Daisuke Ueshima, Keiichi Hishikari, Hirofumi Hioki, Yusuke Watanabe, Kazuki Mizutani, Yae Matsuo, Kentaro Hayashida, Kentaro Jujo, Gaku Nakazawa, SUNRISE Lab Investigators

**Affiliations:** a Department of Cardiology, Teikyo University School of Medicine, Tokyo, Japan; b Department of Cardiology, Japanese Red Cross Ashikaga Hospital, Ashikaga, Japan; c Department of Cardiovascular Medicine, Asahi General Hospital, Asahi, Japan; d Department of Cardiovascular Medicine, St. Luke’s International Hospital, St. Luke's International University, Tokyo, Japan; e Department of Cardiology, Tokai University School of Medicine, Isehara, Japan; f Department of Cardiology, Kameda Medical Center, Kamogawa, Japan; g Department of Cardiology, Cardiovascular Center, Yokosuka Kyosai Hospital, Yokosuka, Japan; h Department of Cardiology, Kindai University Faculty of Medicine, Osaka, Japan; i Department of Cardiovascular Medicine, Cardiovascular Hospital of Central Japan (Kitakanto Cardiovascular Hospital), Shibukawa, Gunma, Japan; j Department of Cardiology, Keio University School of Medicine, Tokyo, Japan; k Department of Cardiology, Saitama Medical University/Saitama Medical Center, Saitama, Japan

**Keywords:** antithrombotic therapy, bleeding risk, transcatheter aortic valve replacement, transcatheter edge-to-edge repair, transcatheter structural heart interventions

## Abstract

Transcatheter structural heart interventions have drastically evolved over the past 2 decades. However, most catheterization procedures require the deployment of devices in the body; therefore, the adhesion of thrombi to those devices is a major problem, resulting in the requirement of a period of postprocedural antithrombotic regimen. However, in recent years, bleeding associated with these antithrombotic therapies has also become a major concern, attracting the attention of investigators. This is complicated by the fact that patients at high thrombotic risk are also at high bleeding risk, making the issue of administrating antithrombotic therapy challenging. The objective of this review was to identify the important issues and summarize the current status of postoperative antithrombotic therapy and assessment of the bleeding risk following transcatheter structural heart interventions such as transcatheter aortic valve replacement, transcatheter edge-to-edge repair, and transcatheter left atrial appendage occlusion.

## Highlights

• The risk of bleeding caused by antithrombotic therapy after transcatheter structural heart interventions has long been a concern, especially for the Asian population.
• This review summarizes the current status of antithrombotic therapy after TAVR, TEER, and LAAO.
• Further studies are ongoing to clarify how to balance between thrombotic and bleeding risks.

Structural heart disease (SHD) is associated with high mortality and morbidity, and the mortality rate increases with age. To date, remarkable progress has been achieved in the treatment of SHD, including medical therapy, open heart surgery, and transcatheter structural heart interventions. Transcatheter structural heart interventions have changed drastically over the past 2 decades, and various procedures can now be performed instead of open heart surgery. Elderly patients tend to prefer catheter-based treatment because it is less invasive than open surgery. However, most catheterization procedures require the deployment of a particular device in the body. Therefore, the adhesion of thrombi to those devices is a major problem in this setting, resulting in the requirement of antithrombotic therapy. As discussed in the following text, guidelines recommend the use of antiplatelet agents and anticoagulants for a certain period of time after almost all percutaneous catheterization procedures. When thromboprophylaxis is the only concern, more aggressive antithrombotic therapy should be continued. However, in recent years, bleeding associated with these antithrombotic therapies has become a major problem, attracting the attention of investigators. This condition is termed high bleeding risk (HBR).^1^ Therefore, the decision regarding the duration and agents of antithrombotic therapy should be made based on an assessment of the bleeding and thrombotic risks in each patient, and requires the provision of careful individualized care. This is complicated by the fact that patients at high thrombotic risk are also at HBR, making the issue of administrating antithrombotic therapy challenging. In addition, although there are defined durations of antithrombotic therapy for transcatheter aortic valve replacement (TAVR), transcatheter edge-to-edge repair (TEER), and transcatheter left atrial appendage occlusion (LAAO) (ie, the most common types of transcatheter structural heart interventions), there is a significant overlap between patient populations. Moreover, few reports have discussed these durations thus far. The objective of this review was to identify the important issues and summarize the current status of postoperative antithrombotic therapy and assessment of the bleeding risk for TAVR, TEER, and LAAO.

## Overview of Bleeding and Thrombotic Events After TAVR

TAVR is effective in the treatment of symptomatic aortic stenosis with broad clinical indications.^2^ Despite its extensive clinical application, TAVR is commonly linked to bleeding and thromboembolic events. Bleeding events after TAVR have been associated with poor clinical outcome, regardless of the time of treatment initiation. Moreover, the presence of life-threatening bleeding is strongly associated with an increased risk of mortality.^3^ In the literature, major and life-threatening bleeding has been observed in 3% to 11% of patients within the first year after TAVR.^4^ Several risk factors of bleeding events after TAVR have been proposed; however, their impact on bleeding events differs depending on the elapsed time since TAVR. In particular, procedural factors (eg, access site and residual aortic regurgitation, periprocedural anticoagulant, postprocedural thrombocytopenia, and acquired von Willebrand syndrome) mainly affect bleeding events during the early phase after TAVR. Notably, advanced age, comorbidities, and chronic antithrombotic therapy are consistently related to a higher incidence of bleeding events regardless of the elapsed time after TAVR.^5^ Because almost all patients undergoing TAVR have either advanced age or several comorbidities, most patients meet the criteria of ARC-HBR.^6^ Based on the currently available studies, the importance of HBR in the ARC-HBR criteria appears to be high in patients undergoing TAVR, particularly in combination with advanced age and chronic kidney disease.^7^

Stroke caused by dislodgment of calcification, plaque, or other foreign material is the most common thrombotic event during and after TAVR. The 30-day and 1-year stroke rates range from 1.0% to 5.5% and 4.3% to 8.2%, respectively.^8^, ^9^, ^10^, ^11^ Similar to the prognostic impact of bleeding events after TAVR, stroke after TAVR is associated with higher mortality rates (30-day mortality: 20%).^12^ The presence of thromboembolic conditions has been reported as a risk factor of stroke after TAVR.^13^ Acute coronary syndrome is another important sequela of thrombotic events. Approximately 10% of patients undergoing TAVR experienced acute coronary syndrome after TAVR at a median follow-up of 25 months; male sex, prior history of coronary artery disease, and the nontransfemoral approach were highly associated with the development of acute coronary syndrome.^14^ Although the incidence of systemic embolization remains unknown, recent randomized clinical trials revealed that 0.1% to 0.3% of patients who underwent TAVR experienced systemic embolization.^15^ Notably, there is a decreasing trend in the incidence rates of both bleeding and thrombotic events as the clinical indication of TAVR shifts from high risk to intermediate or low risk.

## Valve (Leaflet) Thrombosis

Following the first report of leaflet thrombosis after TAVR, several studies have attempted to determine its precise incidence and impact on clinical practice.^16^ Generally, symptomatic valve (leaflet) thrombosis after TAVR is relatively rare (0.6%-2.8%) and can be resolved by anticoagulation.^17^ In contrast, subclinical leaflet thrombosis (SLT) exhibits a markedly higher incidence rate (10%-15%) and is rarely associated with clinical outcome.^18^ It remains unclear whether the presence of SLT affects clinical events (stroke and/or structural valve deterioration). Interestingly, registry data demonstrated that SLT is related to a higher incidence of transient ischemic attack, but not stroke.^18^ SLT may progress to hypoattenuated leaflet thickening and more severe reduced leaflet motion (RLM), which may cause an increase in the transvalvular gradient.^18^ The selection of the most appropriate antithrombotic regimen in patients with SLT remains a concern. Vitamin K anticoagulants (VKAs) and direct oral anticoagulants (DOACs) may protect against leaflet thrombosis. It has been reported that SLT regresses even without changing the antithrombotic regimen.^19^ Moreover, the GALILEO-4D (Global Study Comparing a Rivaroxaban-based Antithrombotic Strategy to an Antiplatelet-based Strategy After TAVR to Optimize Clinical Outcomes) trial demonstrated higher mortality rate in the DOAC group despite the decreased incidence of RLM.^20^ Thus, the routine use of anticoagulation as postprocedural antithrombotic therapy is not currently recommended, especially in elderly patients undergoing TAVR with a higher bleeding tendency.

## American vs European vs Japanese Recommendation in Antithrombotic Therapy After TAVR

According to the American guidelines published in 2020, for patients with a bioprosthetic TAVR who are at low risk of bleeding, dual antiplatelet therapy (DAPT) with aspirin 75 to 100 mg and clopidogrel 75 mg or oral anticoagulant (OAC) with VKA may be reasonable for at least 3 months after TAVR (Class IIb).^21^ Single antiplatelet therapy (SAPT) with aspirin is reasonable in the absence of other indications for OAC (Class IIa).^21^ According to the latest ESC guidelines published in 2021, lifelong treatment with OAC is recommended after TAVR in patients with other indications for OAC (Class I).^22^ Moreover, lifelong SAPT is recommended after TAVR in patients without baseline indication for OAC (Class I).^22^ Routine use of OAC is not recommended after TAVR in patients without baseline indication for OAC (Class III).^22^

In contrast, the current Japanese guidelines recommend DAPT after TAVR for 6 months regardless of the patient’s bleeding risk, and there is no description concerning the indication for OAC.^23^ The Japanese guidelines for valvular heart disease should be updated based on the previous randomized control trials (RCTs) to more robustly define and refine the optimal use of TAVR in patients with severe aortic stenosis. In addition, we suggest that the antithrombotic regimen should be stratified based on various factors. Therefore, further research with longer follow-up is needed to determine the most appropriate antithrombotic regimen in patients undergoing TAVR with and without an indication for OAC.

## Recent Evidence on Antithrombotic Therapy After TAVR

Antithrombotic management should differ between patients with and without an established indication for OAC. The major RCTs investigating the optimal antithrombotic therapy after TAVR are summarized in Table 1.

**Table 1:** Major Randomized Controlled Trials Investigating Optimal Antithrombotic Therapy After TAVR

Trial	ATLANTIS^26^	GALILEO	ENVISAGE-TAVI AF^25^	POPular-TAVI^28^
ClinicalTrials.gov identifier	NCT02664649	NCT02556203	NCT02943785	NCT02247128
Experimental arm	Apixaban 5 mg twice daily	Rivaroxaban 10 mg once daily plus acetylsalicylic acid 75-100 mg (for 90 d only)	Edoxaban 60 mg	Cohort A: aspirin plus clopidogrel; Cohort B: VKA plus clopidogrel
Comparison arm	VKA or antiplatelet therapy or combination	Aspirin and clopidogrel for 3 mo only	VKA	Cohort A: aspirin only; Cohort B: VKA only
Patient number	Stratum 1 (indication for OAC): 228 Stratum 2 (no indication for OAC): 523	1,644	1,426	Cohort A: 665 Cohort B: 313
Design	Randomized, multicenter, open-label	Event-driven, randomized, multicenter, open-label	Event-driven, randomized, multicenter, open-label	Randomized, multicenter, open-label
Nonvalvular atrial fibrillation patients	Included and stratified for	Excluded	Included	Included and stratified for
Follow-up, months	13	17	36	12
Primary endpoint (composite of)	Death, myocardial infarction, stroke, systemic embolism, intracardiac or bioprosthesis thrombus, any episode of deep vein thrombosis or pulmonary embolism, life-threatening or disabling or major bleeding defined according to VARC-2 definitions over 1-y follow-up	All-cause death, myocardial infarction, stroke, systemic embolism, symptomatic valve thrombosis, deep vein thrombosis or pulmonary embolism; composite of adjudicated life-threatening, disabling or major bleeding, classified according to the VARC definitions following the BARC classification	Primary efficacy endpoint: death, myocardial infarction, ischemic stroke, systemic embolic events, valve thrombosis, and major bleeding per definition of the International Society on Thrombosis and Hemostasis Primary safety endpoint: major bleeding	Freedom from all bleeding complications at 1 y after TAVR (coprimary outcome: freedom of nonprocedure related bleeding complications)
Conclusion of the trials	Stratum 1: Insufficient evidence of superiority for the primary endpoint Stratum 2: Insufficient evidence of superiority for the primary endpoint	The trial was terminated prematurely by the data and safety monitoring board because of safety concerns.	Efficacy: Noninferiority for the primary endpoint Safety: Insufficient evidence of noninferiority for the primary endpoint	Cohort A: Superiority for the primary endpoint Cohort B: Superiority for the primary endpoint

ATLANTIS = Anti-Thrombotic Strategy to Lower All Cardiovascular and Neurologic Ischemic and Hemorrhagic Events after Trans-Aortic Valve Replacement for Aortic Stenosis; BARC = Bleeding Academic Research Consortium; ENVISAGE-TAVI AF = Edoxaban Compared to Standard Care After Heart Valve Replacement Using a Catheter in Patients With Atrial Fibrillation; GALILEO = Global Study Comparing a Rivaroxaban-based Antithrombotic Strategy to an Antiplatelet-based Strategy After TAVR to Optimize Clinical Outcomes; OAC = oral anticoagulant; POPular-TAVR = Antiplatelet Therapy for Patients Undergoing Transcatheter Aortic Valve Replacement; TAVR = transcatheter aortic valve replacement; VAK = vitamin K antagonist; VARC = Valve Academic Research Consortium.

We demonstrated the long-term efficacy of DOAC compared with VKA in patients with atrial fibrillation who were successfully discharged after TAVR.^24^ The POPular-TAVR (Antiplatelet Therapy for Patients Undergoing Transcatheter Aortic Valve Replacement) Cohort B trial demonstrated that, in patients undergoing TAVR who were receiving OAC (patients with AF: 95%), the incidence of serious bleeding over a period of 1 month or 1 year was lower with OAC alone vs OAC plus clopidogrel.^4^ Likewise, the randomized ENVISAGE-TAVR AF (Edoxaban Compared to Standard Care After Heart Valve Replacement Using a Catheter in Patients With Atrial Fibrillation) trial showed that, in patients with mainly prevalent AF who underwent successful TAVR, edoxaban was noninferior to VKA.^25^ This was determined by an HR margin of 38% for a composite primary outcome of adverse clinical events, although the incidence of major bleeding was higher with edoxaban than VKA.^25^ These findings suggest that DOAC could be used in patients with AF undergoing TAVR for whom treatment with an OAC is indicated. In contrast, the stratum 1 of the randomized ATLANTIS (Anti-Thrombotic Strategy to Lower All Cardiovascular and Neurologic Ischemic and Hemorrhagic Events after Trans-Aortic Valve Replacement for Aortic Stenosis) trial demonstrated insufficient evidence of superiority for the primary endpoint because of insufficient statistical power for this comparison.^26^

In patients without an established indication for OAC, the stratum 2 of ATLANTIS can be viewed as a trial of DOAC vs antiplatelet therapy, albeit without sufficient statistical power.^26^ From this perspective, it shares with the GALILEO trial the failure of a non-vitamin K OAC-based strategy to reduce the primary outcome, although without evidence of harm caused by apixaban.^27^ Although these 2 trials targeted a similar TAVR population, the intervention differed for several reasons. First, beyond testing a different non-vitamin K OAC (apixaban instead of rivaroxaban), ATLANTIS used the recommended dose for the prevention of cardioembolic stroke in patients with nonvalvular AF, whereas GALILEO explored low-dose rivaroxaban (10 mg daily) along with aspirin. Second, the control strategy differed. GALILEO used 3-month DAPT followed by aspirin alone, whereas the control group in ATLANTIS consisted of SAPT/DAPT according to local practice. Third, the primary endpoint in GALILEO was a composite of all-cause death or thromboembolic events, whereas the primary endpoint in ATLANTIS also included bleeding events. The high rate of DAPT in the control group of ATLANTIS probably contributed to the lower ischemic outcomes and increase in bleeding, thereby affecting the treatment effect of apixaban. Consistent with GALILEO, ATLANTIS demonstrated a higher rate of noncardiovascular death with the experimental strategy. However, such events were mainly caused by sepsis or end-stage renal disease, without any corresponding increase in bleeding or ischemic complications. The POPular-TAVI trial (Cohort A) demonstrated that, among patients undergoing TAVR without an indication for OAC, the incidence of bleeding and the composite of bleeding or thromboembolic events at 1 year were significantly less frequent with SAPT (aspirin) vs DAPT (aspirin plus clopidogrel) administered for 3 months.^28^ Recently, compared with SAPT/DAPT, the nonantiplatelet therapy was not associated with an increased risk of the composite of thromboembolic and bleeding events and potentially reduced the risk of bleeding events in patients undergoing TAVR.^29^ DAPT could only be administered in patients undergoing TAVR with recent percutaneous coronary intervention (PCI) or EVT and no indication for OAC.

Finally, our team recently demonstrated that monotherapy with clopidogrel was associated with a lower incidence of cardiovascular death vs monotherapy with aspirin during a 2-year follow-up after TAVR regardless of anticoagulation use.^30^ This evidence should be investigated in a future RCT.

## Unmet Need for Post-TAVR Antithrombotic Therapy

In patients who underwent TAVR without indication for OAC, the need for antiplatelet therapy to avoid future thrombotic events remains an unresolved issue. To fulfill such unmet clinical needs in antithrombotic therapy, future trials of aspirin vs clopidogrel (prasugrel) as well as nonantiplatelet therapy vs SAPT (ie, aspirin, clopidogrel, or prasugrel) in patients without indication for OAC would be required. According to the current concept, disruption of the endothelial layer covering the fibrosa promotes the uptake of oxidatively modified lipids (along with the protein-cargo they carry), red blood cells, and immune cells, thereby promoting an inflammation-calcification feedback loop that results in AS. From this point of view, antiplatelet therapy as secondary prevention of calcific aortic valve disease progression may not be mandatory. Furthermore, there have been profound changes and improvements in medical therapy with numerous new therapeutic agents, such as high-intensity statin, proprotein convertase subtilisin/kexin type 9 inhibitors, more powerful P2Y_12_ inhibitors, low-dose rivaroxaban, and newer diabetic agents, which continue to improve the overall prognosis of patients. Establishment of a tailored optimal medical therapy regimen following TAVR in parallel with the expanding indication for TAVR into wider and younger populations is important.

Current evidence on transcatheter heart valve hemocompatibility is limited by several factors. First, the resolution of multidetector computed tomography (MDCT) remains an important limitation for the accurate assessment of leaflet mobility and SLT, which is characterized by hypoattenuated leaflet thickening and RLM observed on 4-dimensional computed tomography imaging. Second, interobserver variability in the detection of SLT is high. Furthermore, the timings at which MDCT was performed after TAVR varied widely among different studies. Likewise, the definitions of SLT differed between studies. Earlier data showed a higher incidence of SLT in the balloon-expandable valve and intra-annular position compared with the self-expanding valve and supra-annular position.^16^ Another challenge in the interpretation of SLT is the sudden emergence and spontaneous resolution of some of these lesions. This phenomenon should be taken into consideration when designing subanalyses with MDCT in future trials.

## Antithrombotic Therapy After Transcatheter Edge-To-Edge Mitral Valve Repair

Antithrombotic management after TEER remains debatable. To date, there is no mandatory rule to use anticoagulation after the intervention worldwide.

An incidence of 0.9% of ischemic stroke was documented at 30-day follow-up in EVEREST (Endovascular Valve Edge-to-Edge Repair Study),^31^ 2.6% in the EVEREST High-Risk Registry, and 2.4% in EVEREST REALISM (Real World Expanded Multicenter Study of the Mitra-Clip System).^32^ Currently, there is no recommendation of antithrombotic therapy for patients undergoing TEER in the latest American and European guidelines.^21^^,^^22^ Furthermore, no studies are examining the long-term effects of antithrombotic agents in those patients. The most adopted treatment regimen derives from the protocol of clinical studies testing the devices. This generally consists of 1- to 6-month DAPT of aspirin and clopidogrel followed by aspirin alone for 12 months or longer in patients without indication for OAC. Atrial fibrillation develops commonly in patients with mitral regurgitation, with a reported rate as high as 5% per year.^33^ In the COAPT trial, >50% patients had a history of atrial fibrillation or flutter.^34^

Although there is no recommendation of antithrombotic therapy after TEER in the current Japanese guidelines, our recommendation is summarized in Table 2. In our daily practice, SAPT is chosen in patients undergoing isolated TEER without indication for OAC. DAPT followed by SAPT is chosen in patients undergoing TEER and PCI, and the duration of DAPT is depending on PCI. Furthermore, OAC alone is chosen in atrial fibrillation patients after TEER. In patients after TEER who were receiving OAC, OAC alone might be a good choice to decrease the incidence of serious bleeding events.

**Table 2:** Recommendation of Antithrombotic Regimens in Patients Undergoing TAVR, TEER, and LAAO

Patients With	TAVR	TEER	LAAO (AF)
	SAPT	SAPT	OAC → SAPT (at 45-d follow-up after echocardiographic documentation of effective closure of the appendage)
+ PCI	DAPT (depending on PCI) → SAPT	DAPT (depending on PCI) → SAPT	SAPT + OAC → SAPT (at 45-d follow-up after echocardiographic documentation of effective closure of the appendage)
+ AF	OAC	OAC	NA
+ PCI + AF	SAPT + OAC (depending on PCI) → OAC	SAPT + OAC (depending on PCI) → OAC	NA

AF = atrial fibrillation; DAPT = dual antiplatelet therapy; LAAO = left atrial appendage occlusion; PCI = percutaneous coronary intervention; SAPT = single antiplatelet therapy; TEER = transcatheter edge-to-edge repair; other abbreviations as in Table 1.

## Antithrombotic Therapy After Transcatheter Left Atrial Appendage Occlusion

To date, the optimal antithrombotic therapy in patients undergoing transcatheter LAAO remains debatable. This is mainly related to the difficulties in balancing ischemic and bleeding risk in a frail population with advanced age, multiple comorbidities, cognitive impairment, and clinically relevant unsteadiness gait.^35^

Two landmark trials, PROTECT-AF (Watchman Left Atrial Appendage System for Embolic Protection in Patients With Atrial Fibrillation) and PREVAIL (Watchman LAA Closure Device in Patients With Atrial Fibrillation Versus Long Term Warfarin Therapy), compared this concept using the WATCHMAN device (Boston Scientific, Marlborough, Massachusetts) against warfarin in patients who were candidates for long-term OAC.^36^, ^37^, ^38^, ^39^ In PROTECT-AF and PREVAIL, OAC was continued for at least 45 days after LAAO, followed by transesophageal echocardiography. OAC was replaced by antiplatelet therapy after echocardiographic documentation of effective closure of the appendage, whereas patients were recommended to continue OAC in case of incomplete closure with a peridevice leak >5 mm. Although OAC may be a better therapy to protect against device-related thrombosis during endothelialization in the initial phase after LAAO, it may associate with a higher bleeding rate than antiplatelet drugs. Patients from the PROTECT-AF,^36^, ^37^, ^38^ PREVAIL,^39^ CAP (Continued Access to PROTECT-AF), CAP2 (Continued Access to PREVAIL), ASAP (ASA Plavix Feasibility Study With Watchman Left Atrial Appendage Closure Technology),^40^ and EWOLUTION (Registry on WATCHMAN Outcomes in Real-Life Utilization)^41^ trials receiving either OAC or antiplatelet therapy postimplantation were matched and compared for nonprocedural bleeding and stroke or systemic thromboembolism over 6 months following implantation. The 6-month freedom from nonprocedural major bleeding was similar (OAC 95.7%; antiplatelet therapy 95.5%; *P =* 0.775) despite more early bleedings with OAC.^42^ Freedom from thromboembolism beyond 7 days was similar between groups (OAC 98.8%; antiplatelet therapy 99.4%; *P =* 0.089).^42^ However, device-related thrombosis was more frequent with antiplatelet therapy (OAC 1.4%; antiplatelet therapy 3.1%; *P =* 0.018).^42^

In the LAAO Registry of the National Cardiovascular Data Registry in the United States, among 31,994 patients undergoing successful LAAO between 2016 and 2018, only 12.2% of the patients received the antithrombotic protocol used in the pivotal trials. The most common antithrombotic regimens at discharge were VKA and aspirin (36.9%), DOAC and aspirin (20.8%), followed by VKA alone (13.5%), DOAC alone (12.3%), and DAPT (5.0%). The risk of adverse events at the 45-day follow-up were significantly lower with VKA or DOAC alone compared with VKA and aspirin.^43^

In the PCI field, the “East Asian paradox” has been described, in which East Asian patients have a similar or even a lower rate of ischemic events compared with white patients.^44^ We believe that this phenomenon is referred to in the structural heart intervention field. Therefore, extrapolation of major trials of LAAO, as well as guidelines based on these trials, might not be applicable to East Asian patients. According to the Japanese guidelines, LAAO is recommended as Class IIb, Level of Evidence: B to reduce the risk of stroke in patients with atrial fibrillation who are not suitable candidates for long-term anticoagulation.^45^ In such patients, we suggest that OAC alone (DOAC or VKA) followed by SAPT (aspirin or clopidogrel) at the 45-day follow-up after echocardiographic documentation of effective closure of the appendage. Furthermore, LAAO after other structural heart interventions (TAVR or TEER) may become a standard care for HBR or high thrombotic risk patients with atrial fibrillation in near future (Central Illustration). LAAO could play an important role in preventing stroke instead of anticoagulant in patients with atrial fibrillation.

**Central Illustration:**
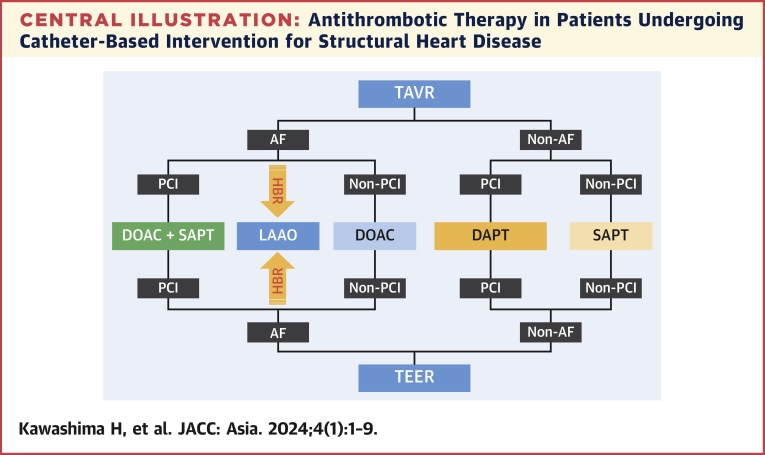
Antithrombotic Therapy in Patients Undergoing Catheter-Based Intervention for Structural Heart Disease As there is significant overlap among patients with structural heart disease, a patient-specific antithrombotic regimen should be considered. AF = atrial fibrillation; DAPT = dual antiplatelet therapy; DOAC = direct oral anticoagulant; HBR = high bleeding risk; LAAO = left atrial appendage occlusion; PCI = percutaneous coronary intervention; SAPT = single antiplatelet therapy; TAVR = transcatheter aortic valve replacement; TEER = transcatheter edge-to-edge repair.

### Summary

SHD is associated with high mortality and morbidity. Moreover, bleeding complications are strongly correlated with poor prognosis. Currently, there are various catheterization therapies available for SHD using valve prostheses, balloons, and other devices. Most patients who require those less-invasive catheter-based therapies are older and/or have several comorbidities associated with HBR, such as anemia, low body weight, chronic kidney disease, and frailty. In addition, there is significant overlap among patients with transcatheter structural heart interventions (Central Illustration). Therefore, a patient-based, tailor-made antithrombotic regimen considering the HBR is expected. Although there are guidelines or consensus documents for each interventional procedure, there are no comprehensive statements covering TAVR, TEER, and LAAO. Notably, the important determining step for the antithrombotic regimen is any indication for OAC in patients undergoing any catheter-based procedures. This review discusses these complex issues and summarizes the current status of postoperative antithrombotic therapy and bleeding risk assessment for TAVR, TEER, and LAAO. However, there are also many other transcatheter procedures, such as atrial septal detect, ventricular septal detect, and paravalvular leakage closure, that definitely need ongoing studies and patient-directed individual treatment strategies to balance between antithrombotic effect and bleeding, which lead to improvement in the quality of care.

## Funding Support and Author Disclosures

Drs Watanabe, Mizutani, and Hayashida are clinical proctors for Edwards Lifesciences and Medtronic. Dr Nakazawa is a consultant for Boston Scientific, Terumo Corp, OrbusNeich Medical, and Japan Medical Device Technology Co, Ltd; and has received lecture fees from Abbott Medical and Terumo Corp. All other authors have reported that they have no relationships relevant to the contents of this paper to disclose.
